# Supplementary figures and images for: Low-dose theophylline in addition to ICS therapy in COPD patients: A systematic review and meta-analysis
Source: PLoS One. 2021 May 24;16(5):e0251348. doi: 10.1371/journal.pone.0251348 (PMC8143407; doi:10.1371/journal.pone.0251348)

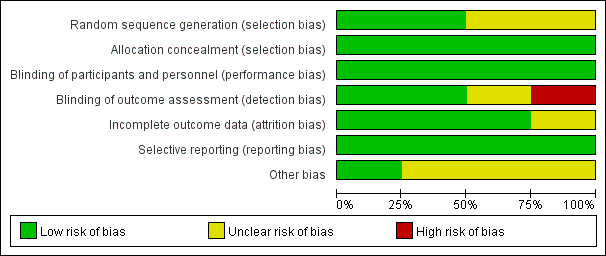

Supplement: S1 Fig — (TIF) [file pone.0251348.s001.tif]

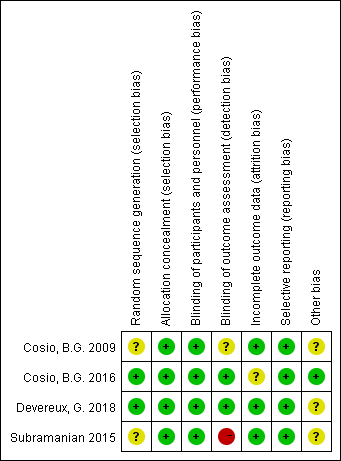

Supplement: S2 Fig — (TIF) [file pone.0251348.s002.tif]

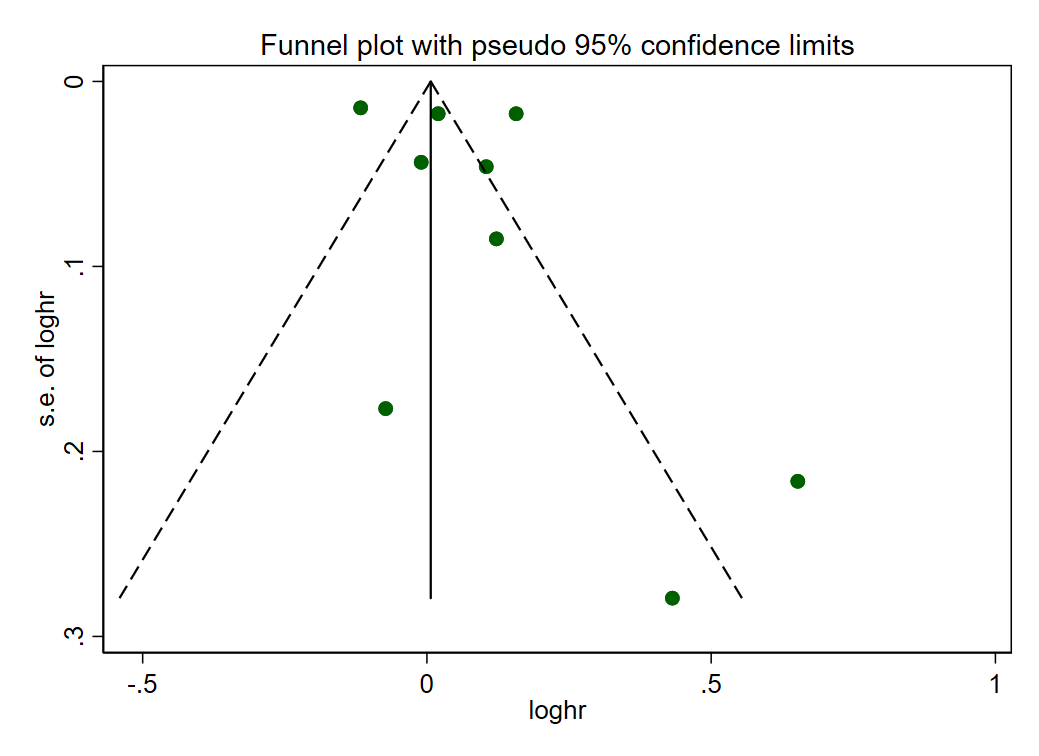

Supplement: S3 Fig — (TIF) [file pone.0251348.s003.tif]

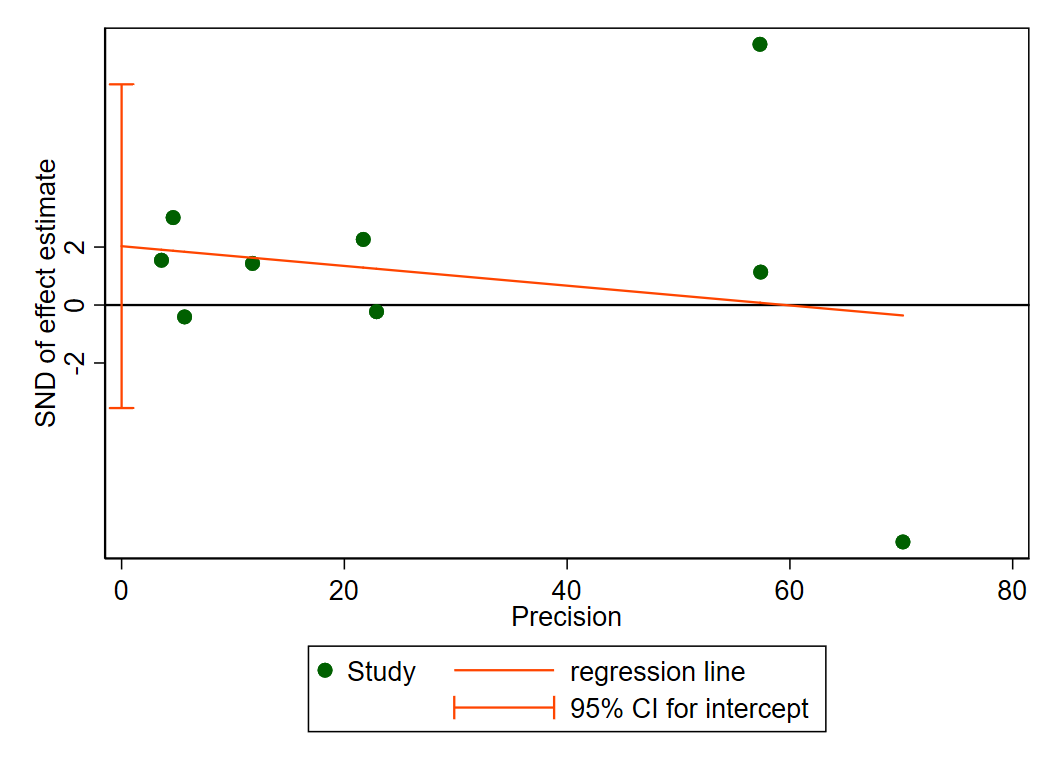

Supplement: S4 Fig — (TIF) [file pone.0251348.s004.tif]
