# Supplementary material for: Low-dose theophylline in addition to ICS therapy in COPD patients: A systematic review and meta-analysis
Source: PLoS One. 2021 May 24;16(5):e0251348. doi: 10.1371/journal.pone.0251348 (PMC8143407; doi:10.1371/journal.pone.0251348)
Supplement: S1 File — (DOC) [file pone.0251348.s005.doc]

**Cochrane CENTRAL search strategy**

[Pulmonary Disease, Chronic Obstructive](https://www.cochranelibrary.com/advanced-search/mesh#0)

Synonyms: Airflow Obstruction, Chronic; Chronic Airflow Obstructions; Chronic Airflow Obstruction; Airflow Obstructions, Chronic; COPD; COAD; Chronic Obstructive Pulmonary Disease; Chronic Obstructive Airway Disease; Chronic Obstructive Lung Disease

[Lung Diseases, Obstructive](https://www.cochranelibrary.com/advanced-search/mesh#0)

Synonyms: Obstructive Pulmonary Disease; Pulmonary Disease, Obstructive; Lung Disease, Obstructive; Obstructive Lung Disease; Obstructive Pulmonary Diseases; Pulmonary Diseases, Obstructive; Obstructive Lung Diseases

[Adrenal Cortex Hormones](https://www.cochranelibrary.com/advanced-search/search-manager#0)

Synonyms: Corticoids; Corticosteroids; Hormones, Adrenal Cortex

[Glucocorticoids](https://www.cochranelibrary.com/advanced-search/search-manager#0)

Synonyms: Glucocorticoid; Glucocorticoid Effect; Effects, Glucorticoid; Effect, Glucocorticoid; Glucorticoid Effects

[Theophylline](https://www.cochranelibrary.com/advanced-search/search-manager#0)

Synonyms: 3,7-Dihydro-1,3-dimethyl-1H-purine-2,6-dione; 1,3-Dimethylxanthine; 1,3 Dimethylxanthine; Theocin; Somophyllin T; Somophyllin-T; SomophyllinT; Glycine Theophyllinate; Theophyllinate, Glycine; Theodur; Theo-Dur; Theo Dur; Afonilum Retard; Theo Von Ct; Ct, Theo Von; Von Ct, Theo; Quibron T SR; Quibron T-SR; Quibron TSR; Theonite; Uniphylline; Elixophyllin; Bronkodyl; Uniphyl; Uniphyllin; Bronchoparat; Anhydrous, Theophylline; Theophylline Anhydrous; Slo Phyllin; Slo-Phyllin; SloPhyllin; Theovent; Armophylline; Nuelin; Nuelin S.A.; Theolair; Sustaire; Theostat; Lodrane; Theobid; Theospan; Aerobin; Synophylate; Euphylong; Sodium Glycinate, Theophylline; Theophylline Sodium Glycinate; Glycinate, Theophylline Sodium; Aquaphyllin; Theon; Constant T; Constant-T; ConstantT; Theo 24; Theo24; Theo-24; Theoconfin Continuous; Monospan; Theolix; Accurbron; Theopek; Aerolate

[Aminophylline](https://www.cochranelibrary.com/advanced-search/search-manager#0)

Synonyms: Euphyllin Retard; Mundiphyllin Retard; Diaphyllin; Tari-Dog; Euphyllin; Eufilina; Aminodur; Cardophyllin; Clonofilin; Euphylline; Somophyllin; Eufilina Venosa; Drafilyn; Mini-Lix; Phyllotemp; Mundiphyllin; Truphylline; Novophyllin; Theophyllamin Jenapharm; Aminophylline DF; Theophyllamine; Theophylline Ethylenediamine; Ethylenediamine, Theophylline; Duraphyllin; Theophyllin EDA ratiopharm; Theophyllin EDA-ratiopharm; Theophyllin EDAratiopharm; Aminophyllin; Afonilum; Carine; Godafilin; Phyllocontin; Corophyllin

Search Name: T-C-C

Date Run: 21/08/2019 22:43:52

Comment:

ID Search Hits

#1 MeSH descriptor: [Pulmonary Disease, Chronic Obstructive] explode all trees 4833

#2 (Airflow Obstruction, Chronic):ti,ab,kw OR (Chronic Airflow Obstructions):ti,ab,kw OR (Chronic Airflow Obstruction):ti,ab,kw OR (Airflow Obstructions, Chronic):ti,ab,kw OR (COPD):ti,ab,kw (Word variations have been searched) 15033

#3 (COAD):ti,ab,kw OR (Chronic Obstructive Pulmonary Disease):ti,ab,kw OR (Chronic Obstructive Airway Disease):ti,ab,kw OR (Chronic Obstructive Lung Disease):ti,ab,kw 14056

#4 #1 OR #2 OR #3 19337

#5 MeSH descriptor: [Lung Diseases, Obstructive] explode all trees 17975

#6 (Obstructive Pulmonary Disease):ti,ab,kw OR (Pulmonary Disease, Obstructive):ti,ab,kw OR (Lung Disease, Obstructive):ti,ab,kw OR (Obstructive Lung Disease):ti,ab,kw OR (Obstructive Pulmonary Diseases):ti,ab,kw 14480

#7 (Pulmonary Diseases, Obstructive):ti,ab,kw OR (Obstructive Lung Diseases):ti,ab,kw 4053

#8 #5 OR #6 OR #7 26610

#9 #4 OR #8 31477

#10 MeSH descriptor: [Glucocorticoids] explode all trees 4311

#11 (Glucocorticoid):ti,ab,kw OR (Glucocorticoid Effect):ti,ab,kw OR (Effects, Glucorticoid):ti,ab,kw OR (Effect, Glucocorticoid):ti,ab,kw OR (Glucorticoid Effects):ti,ab,kw 3507

#12 #10 OR #11 7048

#13 MeSH descriptor: [Adrenal Cortex Hormones] explode all trees 13818

#14 (Corticoids):ti,ab,kw OR (Hormones, Adrenal Cortex):ti,ab,kw OR (Corticosteroids):ti,ab,kw 13583

#15 #13 OR #14 23986

#16 #12 OR #15 26259

#17 MeSH descriptor: [Theophylline] explode all trees 1723

#18 (1,3 Dimethylxanthine):ti,ab,kw OR (Theocin):ti,ab,kw OR (Somophyllin T):ti,ab,kw OR (Somophyllin-T):ti,ab,kw OR (SomophyllinT):ti,ab,kw 5

#19 (Glycine Theophyllinate):ti,ab,kw OR (Theophyllinate, Glycine):ti,ab,kw OR (Theodur):ti,ab,kw OR (Theo-Dur):ti,ab,kw OR (Theo Dur):ti,ab,kw 126

#20 (Afonilum Retard):ti,ab,kw OR (Theo Von Ct):ti,ab,kw OR (Ct, Theo Von):ti,ab,kw OR (Von Ct, Theo):ti,ab,kw OR (Quibron T SR):ti,ab,kw 5

#21 (Quibron T-SR):ti,ab,kw OR (Quibron TSR):ti,ab,kw OR (Theonite):ti,ab,kw OR (Uniphylline):ti,ab,kw OR (Elixophyllin):ti,ab,kw 13

#22 (Bronkodyl):ti,ab,kw OR (Uniphyl):ti,ab,kw OR (Uniphyllin):ti,ab,kw OR (Bronchoparat):ti,ab,kw OR (Anhydrous, Theophylline):ti,ab,kw 92

#23 (Theophylline Anhydrous):ti,ab,kw OR (Slo Phyllin):ti,ab,kw OR (Slo-Phyllin):ti,ab,kw OR (SloPhyllin):ti,ab,kw OR (Theovent):ti,ab,kw 55

#24 (Armophylline; Nuelin):ti,ab,kw OR (Nuelin S.A.):ti,ab,kw OR (Theolair):ti,ab,kw OR (Sustaire):ti,ab,kw OR (Theostat):ti,ab,kw 21

#25 (Lodrane):ti,ab,kw OR (Theobid):ti,ab,kw OR (Theospan):ti,ab,kw OR (Aerobin):ti,ab,kw OR (Synophylate):ti,ab,kw 4

#26 (Euphylong):ti,ab,kw OR (Sodium Glycinate, Theophylline):ti,ab,kw OR (Theophylline Sodium Glycinate):ti,ab,kw OR (Glycinate, Theophylline Sodium):ti,ab,kw OR (Aquaphyllin):ti,ab,kw 27

#27 (Theon):ti,ab,kw OR (Constant T):ti,ab,kw OR (Constant-T):ti,ab,kw OR (ConstantT):ti,ab,kw OR (Theo 24):ti,ab,kw 1204

#28 (Theo24):ti,ab,kw OR (Theo-24):ti,ab,kw OR (Theoconfin Continuous):ti,ab,kw OR (Monospan):ti,ab,kw OR (Theolix):ti,ab,kw 12

#29 (Accurbron):ti,ab,kw OR (Theopek):ti,ab,kw OR (Aerolate):ti,ab,kw 3

#30 #17 OR #18 OR #19 OR #20 OR #21 OR #22 OR #23 OR #24 OR #25 OR #26 OR #27 OR #28 OR #29 2995

#31 MeSH descriptor: [Aminophylline] explode all trees 364

#32 (Euphyllin Retard):ti,ab,kw OR (Mundiphyllin Retard):ti,ab,kw OR (Diaphyllin):ti,ab,kw OR (Tari-Dog):ti,ab,kw OR (Euphyllin):ti,ab,kw 35

#33 (Eufilina):ti,ab,kw OR (Aminodur):ti,ab,kw OR (Cardophyllin):ti,ab,kw OR (Clonofilin):ti,ab,kw OR (Euphylline):ti,ab,kw 10

#34 (Somophyllin):ti,ab,kw OR (Eufilina Venosa):ti,ab,kw OR (Drafilyn):ti,ab,kw OR (Mini-Lix):ti,ab,kw OR (Phyllotemp):ti,ab,kw 11

#35 (Mundiphyllin):ti,ab,kw OR (Truphylline):ti,ab,kw OR (Novophyllin):ti,ab,kw OR (Theophyllamin Jenapharm):ti,ab,kw OR (Aminophylline DF):ti,ab,kw 1

#36 (Theophyllamine):ti,ab,kw OR (Theophylline Ethylenediamine):ti,ab,kw OR (Ethylenediamine, Theophylline):ti,ab,kw OR (Duraphyllin):ti,ab,kw OR (Theophyllin EDA ratiopharm):ti,ab,kw 55

#37 (Theophyllin EDA-ratiopharm):ti,ab,kw OR (Theophyllin EDAratiopharm):ti,ab,kw OR (Aminophyllin):ti,ab,kw OR (Afonilum):ti,ab,kw OR (Carine):ti,ab,kw 10

#38 (Godafilin):ti,ab,kw OR (Phyllocontin):ti,ab,kw OR (Corophyllin):ti,ab,kw 32

#39 #31 OR #32 OR #33 OR #34 OR #35 OR #36 OR #37 OR #38 473

#40 #30 OR #39 3050

#41 #9 AND #16 AND #40 114

**EMBASE Search Strategy**

**chronic obstructive lung disease**

chronic airflow obstruction; chronic airway obstruction; chronic obstructive bronchitis; chronic obstructive bronchopulmonary disease; chronic obstructive lung disorder; chronic obstructive pulmonary disease; chronic obstructive pulmonary disorder; chronic obstructive respiratory disease; copd; lung chronic obstructive disease; lung disease, chronic obstructive; lung diseases, obstructive; obstructive lung disease; obstructive lung disease, chronic; obstructive pulmonary disease; obstructive respiratory disease; obstructive respiratory tract disease; pulmonary disease, chronic obstructive; pulmonary disorder, chronic obstructive

corticosteroid

Synonyms

adrenal cortex hormone; adrenal cortex hormones; adrenal cortical hormone; adrenal cortical hormones; adrenal cortical steroid; adrenal steroid; adrenal steroid hormone; adreno cortical steroid; adreno corticosteroid; adrenocortical hormone; adrenocortical steroid; adrenocorticosteroid; cortical steroid; cortico steroid; corticoid; corticosteroid agent; corticosteroid calcium; corticosteroid hormone; corticosteroids; corticosteroids, inhalation; corticosteroids, ophthalmic; corticosteroids, otic; corticosteroids, systemic; corticosteroids, topical; dermocorticosteroid; fluorinated corticosteroid

theophylline

Synonyms

1, 3 dimethylxanthine; accurbron; aerobin; aerodyne retard; aerolate; aerolate iii; aerolate jr; aerolate sr; afonilum; afonilum forte; afonilum mite; afonilum retard; afonilum sr; almarion; aquaphyllin; armophylline; asmasalon; asperal-t; austyn; bilordyl; bronchoretard; broncophyl; bronkodyl; bronkotabs; bronsolvan; by 912; by912; constant t; cronasma; deo-q syrup; dextrose plus theophylline; diffumal; diffumal 24; dilatrane; dilotrane; ditenaten; duraphyl; durofilin; elixicon; elixofilina; elixomin; elixophyllin; elixophyllin sr; elixophylline; eufilina venosa; euphillin; euphyllin; euphyllin cr; euphyllin cr retard; euphyllin retard; euphyllin retard mite; euphylong; euphylong retardkaps; euphylong sr; gtr 80; labid; labophylline; lanophyllin; lasma; microcrystalline theophylline; monospan; nefoben; neulin; neulin sa; neulin-sr; nik 168 tx; nuelin; nuelin depot; nuelin sa; nuelin sr; palaron; pediaphyllin; pediaphylline la; pharphylline; phylobid; physpan; piridasmin retard; planphylline; pro vent; protheo; pulmidur; pulmo timelets; quibron t; quibron t sr; quibron-t; quibron-t/sr; respbid; respicur; rona phyllin; rona slophyllin; ronaphyllin; ronaslophyllin; slo bid; slo bid gyrocaps; slo-bid; slo-bid gyrocaps; slo-phyllin; slo-theo; slobid; slophyllin; sodip phyllin; solosin; solosin retard; solusin; somofillina; somofillina retardo; somophyllin; somophyllin-crt; somophyllin-t; somophylline; somophylline crt; somophyllyn; spophyllin; spophyllin retard; sustaire; synophylate; t-phyl; talofilina; talotren; techniphylline; tedralan (theophylline); teobid; teoclear; teoclear la; teofilina retard; teofillina; teolin 300; teolixir; teolong; teonova; teosona; teotard 350; theo 2; theo 24; theo bid; theo dur; theo pa; theo sr; theo von ct; theo-2; theo-24; theo-bros; theo-dur; theo-time; theobeads; theobid; theobid jr.; theobron; theochron; theocin; theoclear; theoclear l.a.-130; theoclear l.a.-260; theoclear-100; theoclear-200; theoclear-80; theocontin; theodur; theodur sprinkle; theofylline; theograd; theolair; theolair la; theolair plus; theolair retard; theolair s; theolair sr; theolair-sr; theolan; theolin; theolin retard; theolin sr; theolixer; theolixir; theolong; theomax; theopec; theopek; theopexine; theophilline; theophyl; theophyl-225; theophyl-sr; theophyllin; theophylline 0.04% and dextrose 5%; theophylline 0.08% and dextrose 5%; theophylline 0.16% and dextrose 5%; theophylline 0.2% and dextrose 5%; theophylline 0.32% and dextrose 5%; theophylline 0.4% and dextrose 5%; theophylline and dextrose 5%; theophylline anhydrous; theophylline bruneau; theophylline hydrate; theophylline hydrobromide; theophylline in dextrose 5%; theophylline monohydrate; theophylline plus dextrose; theophylline retard; theophylline-sr; theoplus; theoplus retard; theospan; theospirex; theospirex retard; theostan cr; theostat; theostat 300; theostat lp; theotard; theotrim; theovent; theovent la; thiophyllin; thiophylline; tiodilax; truxophyllin; tyrex; uni dur; uni-dur; unicontin; unicontin-400 continus; unidur; unifyl; unifyl retard; unilair; uniphyl; uniphyl cr; uniphyllin; uniphyllin continus; uniphylline; vent retard; xanthium (drug); xantivent

Embase Session Results

No.

Query

Results

#16 #3 AND #6 AND #15 2,322

#15 #7 OR #8 OR #9 OR #10 OR #11 OR #12 OR #13 OR #14 47,007

#14 'theophylline sr':ab,ti OR theoplus:ab,ti OR 'theoplus retard':ab,ti OR theospan:ab,ti OR theospirex:ab,ti OR 'theospirex retard':ab,ti OR 'theostan cr':ab,ti OR theostat:ab,ti OR 'theostat 300':ab,ti OR 'theostat lp':ab,ti OR theotard:ab,ti OR theotrim:ab,ti OR theovent:ab,ti OR 'theovent la':ab,ti OR thiophyllin:ab,ti OR thiophylline:ab,ti OR tiodilax:ab,ti OR truxophyllin:ab,ti OR tyrex:ab,ti OR 'uni dur':ab,ti OR unicontin:ab,ti OR 'unicontin-400 continus':ab,ti OR unidur:ab,ti OR unifyl:ab,ti OR 'unifyl retard':ab,ti OR unilair:ab,ti OR uniphyl:ab,ti OR 'uniphyl cr':ab,ti OR uniphyllin:ab,ti OR 'uniphyllin continus':ab,ti OR uniphylline:ab,ti OR 'vent retard':ab,ti OR (xanthium:ab,ti AND drug:ab,ti) OR xantivent:ab,ti 159

#13 'theolair sr':ab,ti OR theolan:ab,ti OR theolin:ab,ti OR 'theolin retard':ab,ti OR 'theolin sr':ab,ti OR theolixer:ab,ti OR theolixir:ab,ti OR theolong:ab,ti OR theomax:ab,ti OR theopec:ab,ti OR theopek:ab,ti OR theopexine:ab,ti OR theophilline:ab,ti OR theophyl:ab,ti OR 'theophyl 225':ab,ti OR 'theophyl sr':ab,ti OR theophyllin:ab,ti OR ('theophylline 0.04%':ab,ti AND 'dextrose 5%':ab,ti) OR ('theophylline 0.08%':ab,ti AND 'dextrose 5%':ab,ti) OR ('theophylline 0.16%':ab,ti AND 'dextrose 5%':ab,ti) OR ('theophylline 0.2%':ab,ti AND 'dextrose 5%':ab,ti) OR ('theophylline 0.32%':ab,ti AND 'dextrose 5%':ab,ti) OR ('theophylline 0.4%':ab,ti AND 'dextrose 5%':ab,ti) OR (theophylline:ab,ti AND 'dextrose 5%':ab,ti) OR 'theophylline anhydrous':ab,ti OR 'theophylline bruneau':ab,ti OR 'theophylline hydrate':ab,ti OR 'theophylline hydrobromide':ab,ti OR 'theophylline in dextrose 5%':ab,ti OR 'theophylline monohydrate':ab,ti OR 'theophylline plus dextrose':ab,ti OR 'theophylline retard':ab,ti 565

#12 'theo bid':ab,ti OR 'theo pa':ab,ti OR 'theo sr':ab,ti OR 'theo von ct':ab,ti OR 'theo 2':ab,ti OR 'theo 24':ab,ti OR 'theo bros':ab,ti OR 'theo dur':ab,ti OR 'theo time':ab,ti OR theobeads:ab,ti OR theobid:ab,ti OR 'theobid jr':ab,ti OR theobron:ab,ti OR theochron:ab,ti OR theocin:ab,ti OR theoclear:ab,ti OR 'theoclear l.a.-130':ab,ti OR 'theoclear l.a.-260':ab,ti OR 'theoclear 100':ab,ti OR 'theoclear 200':ab,ti OR 'theoclear 80':ab,ti OR theocontin:ab,ti OR theodur:ab,ti OR 'theodur sprinkle':ab,ti OR theofylline:ab,ti OR theograd:ab,ti OR theolair:ab,ti OR 'theolair la':ab,ti OR 'theolair plus':ab,ti OR 'theolair retard':ab,ti OR 'theolair s':ab,ti 307

#11 'sodip phyllin':ab,ti OR solosin:ab,ti OR 'solosin retard':ab,ti OR solusin:ab,ti OR somofillina:ab,ti OR 'somofillina retardo':ab,ti OR somophyllin:ab,ti OR 'somophyllin crt':ab,ti OR 'somophyllin t':ab,ti OR somophylline:ab,ti OR 'somophylline crt':ab,ti OR somophyllyn:ab,ti OR spophyllin:ab,ti OR 'spophyllin retard':ab,ti OR sustaire:ab,ti OR synophylate:ab,ti OR 't phyl':ab,ti OR talofilina:ab,ti OR talotren:ab,ti OR techniphylline:ab,ti OR (tedralan:ab,ti AND theophylline:ab,ti) OR teobid:ab,ti OR teoclear:ab,ti OR 'teoclear la':ab,ti OR 'teofilina retard':ab,ti OR teofillina:ab,ti OR 'teolin 300':ab,ti OR teolixir:ab,ti OR teolong:ab,ti OR teonova:ab,ti OR teosona:ab,ti OR 'teotard 350':ab,ti OR 'theo 2':ab,ti 65

#10 nuelin:ab,ti OR 'nuelin depot':ab,ti OR 'nuelin sa':ab,ti OR 'nuelin sr':ab,ti OR palaron:ab,ti OR pediaphyllin:ab,ti OR 'pediaphylline la':ab,ti OR pharphylline:ab,ti OR phylobid:ab,ti OR physpan:ab,ti OR 'piridasmin retard':ab,ti OR planphylline:ab,ti OR 'pro vent':ab,ti OR protheo:ab,ti OR pulmidur:ab,ti OR 'pulmo timelets':ab,ti OR 'quibron t sr':ab,ti OR 'quibron t':ab,ti OR slophyllin:ab,ti OR respbid:ab,ti OR respicur:ab,ti OR 'rona phyllin':ab,ti OR 'rona slophyllin':ab,ti OR ronaphyllin:ab,ti OR ronaslophyllin:ab,ti OR 'slo bid gyrocaps':ab,ti OR 'slo bid':ab,ti OR 'slo-bid gyrocaps':ab,ti OR 'slo phyllin':ab,ti OR 'slo theo':ab,ti OR slobid:ab,ti 107

#9 dilatrane:ab,ti OR dilotrane:ab,ti OR ditenaten:ab,ti OR duraphyl:ab,ti OR durofilin:ab,ti OR elixicon:ab,ti OR elixofilina:ab,ti OR elixomin:ab,ti OR elixophyllin:ab,ti OR 'elixophyllin sr':ab,ti OR elixophylline:ab,ti OR 'eufilina venosa':ab,ti OR euphillin:ab,ti OR euphyllin:ab,ti OR 'euphyllin cr':ab,ti OR 'euphyllin cr retard':ab,ti OR 'euphyllin retard':ab,ti OR 'euphyllin retard mite':ab,ti OR euphylong:ab,ti OR 'euphylong retardkaps':ab,ti OR 'euphylong sr':ab,ti OR 'gtr 80':ab,ti OR labid:ab,ti OR labophylline:ab,ti OR lanophyllin:ab,ti OR lasma:ab,ti OR 'microcrystalline theophylline':ab,ti OR monospan:ab,ti OR nefoben:ab,ti OR neulin:ab,ti OR 'neulin sa':ab,ti OR 'neulin sr':ab,ti OR 'nik 168 tx':ab,ti 328

#8 '1, 3 dimethylxanthine':ab,ti OR accurbron:ab,ti OR aerobin:ab,ti OR 'aerodyne retard':ab,ti OR aerolate:ab,ti OR 'aerolate iii':ab,ti OR 'aerolate jr':ab,ti OR 'aerolate sr':ab,ti OR afonilum:ab,ti OR 'afonilum forte':ab,ti OR 'afonilum mite':ab,ti OR 'afonilum retard':ab,ti OR 'afonilum sr':ab,ti OR almarion:ab,ti OR aquaphyllin:ab,ti OR armophylline:ab,ti OR asmasalon:ab,ti OR 'asperal t':ab,ti OR austyn:ab,ti OR bilordyl:ab,ti OR bronchoretard:ab,ti OR broncophyl:ab,ti OR bronkodyl:ab,ti OR bronkotabs:ab,ti OR bronsolvan:ab,ti OR 'by 912':ab,ti OR by912:ab,ti OR 'constant t':ab,ti OR cronasma:ab,ti OR 'deo-q syrup':ab,ti OR 'dextrose plus theophylline':ab,ti OR diffumal:ab,ti OR 'diffumal 24':ab,ti 859

#7 'theophylline'/exp 46,031

#6 #4 OR #5 966,748

#5 'adrenal cortex hormone':ab,ti OR 'adrenal cortex hormones':ab,ti OR 'adrenal cortical hormone':ab,ti OR 'adrenal cortical hormones':ab,ti OR 'adrenal cortical steroid':ab,ti OR 'adrenal steroid':ab,ti OR 'adrenal steroid hormone':ab,ti OR 'adreno cortical steroid':ab,ti OR 'adreno corticosteroid':ab,ti OR 'adrenocortical hormone':ab,ti OR 'adrenocortical steroid':ab,ti OR adrenocorticosteroid:ab,ti OR 'cortical steroid':ab,ti OR 'cortico steroid':ab,ti OR corticoid:ab,ti OR 'corticosteroid agent':ab,ti OR 'corticosteroid calcium':ab,ti OR 'corticosteroid hormone':ab,ti OR corticosteroids:ab,ti OR 'corticosteroids, inhalation':ab,ti OR 'corticosteroids, ophthalmic':ab,ti OR 'corticosteroids, otic':ab,ti OR 'corticosteroids, systemic':ab,ti OR 'corticosteroids, topical':ab,ti OR dermocorticosteroid:ab,ti OR 'fluorinated corticosteroid':ab,ti 105,259

#4 'corticosteroid'/exp 953,132

#3 #1 OR #2 147,732

#2 'chronic airflow obstruction':ab,ti OR 'chronic airway obstruction':ab,ti OR 'chronic obstructive bronchitis':ab,ti OR 'chronic obstructive bronchopulmonary disease':ab,ti OR 'chronic obstructive lung disorder':ab,ti OR 'chronic obstructive pulmonary disease':ab,ti OR 'chronic obstructive pulmonary disorder':ab,ti OR 'chronic obstructive respiratory disease':ab,ti OR copd:ab,ti OR 'lung chronic obstructive disease':ab,ti OR 'lung disease, chronic obstructive':ab,ti OR 'lung diseases, obstructive':ab,ti OR 'obstructive lung disease':ab,ti OR 'obstructive lung disease, chronic':ab,ti OR 'obstructive pulmonary disease':ab,ti OR 'obstructive respiratory disease':ab,ti OR 'obstructive respiratory tract disease':ab,ti OR 'pulmonary disease, chronic obstructive':ab,ti OR 'pulmonary disorder, chronic obstructive':ab,ti 108,848

#1 'chronic obstructive lung disease'/exp 124,085

**PUBMED**

Pulmonary Disease, Chronic Obstructive

- COPD
- Chronic Obstructive Pulmonary Disease
- COAD
- Chronic Obstructive Airway Disease
- Chronic Obstructive Lung Disease
- Airflow Obstruction, Chronic
- Airflow Obstructions, Chronic
- Chronic Airflow Obstructions
- Chronic Airflow Obstruction

[Lung Diseases, Obstructive](https://www.ncbi.nlm.nih.gov/mesh/68008173)

- Lung Disease, Obstructive
- Obstructive Lung Disease
- Obstructive Lung Diseases
- Obstructive Pulmonary Diseases
- Obstructive Pulmonary Disease
- Pulmonary Disease, Obstructive
- Pulmonary Diseases, Obstructive

Adrenal Cortex Hormones

- Hormones, Adrenal Cortex
- Corticosteroids
- Corticoids

Theophylline

Entry Terms:

- 3,7-Dihydro-1,3-dimethyl-1H-purine-2,6-dione
- 1,3-Dimethylxanthine
- 1,3 Dimethylxanthine
- Armophylline
- Constant-T
- Constant T
- ConstantT
- Elixophyllin
- Euphylong
- Glycine Theophyllinate
- Theophyllinate, Glycine
- Lodrane
- Uniphyllin
- Nuelin S.A.
- Nuelin
- Monospan
- Quibron T-SR
- Quibron T SR
- Quibron TSR
- Slo-Phyllin
- Slo Phyllin
- SloPhyllin
- Somophyllin-T
- Somophyllin T
- SomophyllinT
- Sustaire
- Synophylate
- Theo Von Ct
- Ct, Theo Von
- Von Ct, Theo
- Theo-24
- Theo 24
- Theo24
- Theobid
- Theocin
- Theoconfin Continuous
- Theodur
- Theo-Dur
- Theo Dur
- Theolair
- Theolix
- Theon
- Aerolate
- Theonite
- Theopek
- Theophylline Sodium Glycinate
- Glycinate, Theophylline Sodium
- Sodium Glycinate, Theophylline
- Theospan
- Theostat
- Theovent
- Uniphyl
- Uniphylline
- Theophylline Anhydrous
- Anhydrous, Theophylline
- Accurbron
- Aerobin
- Afonilum Retard
- Aquaphyllin
- Bronchoparat
- Bronkodyl

[Aminophylline](https://www.ncbi.nlm.nih.gov/mesh/68000628)

- Theophylline Ethylenediamine
- Ethylenediamine, Theophylline
- Theophyllamine
- Phyllotemp
- Mundiphyllin
- Theophyllamin Jenapharm
- Theophyllin EDA-ratiopharm
- Theophyllin EDA ratiopharm
- Theophyllin EDAratiopharm
- Truphylline
- Afonilum
- Carine
- Eufilina
- Euphyllin
- Aminodur
- Aminophyllin
- Aminophylline DF
- Cardophyllin
- Clonofilin
- Corophyllin
- Diaphyllin
- Drafilyn
- Duraphyllin
- Eufilina Venosa
- Euphyllin Retard
- Euphylline
- Godafilin
- Mini-Lix
- Mundiphyllin Retard
- Novophyllin
- Phyllocontin
- Somophyllin
- Tari-Dog

| Search | Add to builder | Query | Items found | Time |
| --- | --- | --- | --- | --- |
| [#53](https://www.ncbi.nlm.nih.gov/pubmed/advanced) | [Add](https://www.ncbi.nlm.nih.gov/pubmed/advanced) | Search **(((((("Theophylline"[Mesh]) OR (((((((((((((((((((((((((((((((((((((((((((((((((((((((((((((3,7-Dihydro-1,3-dimethyl-1H-purine-2,6-dione[Title/Abstract]) OR 1,3-Dimethylxanthine[Title/Abstract]) OR 1,3 Dimethylxanthine[Title/Abstract]) OR Armophylline[Title/Abstract]) OR Constant-T[Title/Abstract]) OR Constant T[Title/Abstract]) OR ConstantT[Title/Abstract]) OR Elixophyllin[Title/Abstract]) OR Euphylong[Title/Abstract]) OR Glycine Theophyllinate[Title/Abstract]) OR Theophyllinate, Glycine[Title/Abstract]) OR Lodrane[Title/Abstract]) OR Uniphyllin[Title/Abstract]) OR Nuelin S.A.[Title/Abstract]) OR Nuelin[Title/Abstract]) OR Monospan[Title/Abstract]) OR Quibron T-SR[Title/Abstract]) OR Quibron T SR[Title/Abstract]) OR Quibron TSR[Title/Abstract]) OR Slo-Phyllin[Title/Abstract]) OR Slo Phyllin[Title/Abstract]) OR SloPhyllin[Title/Abstract]) OR Somophyllin-T[Title/Abstract]) OR Somophyllin T[Title/Abstract]) OR SomophyllinT[Title/Abstract]) OR Sustaire[Title/Abstract]) OR Synophylate[Title/Abstract]) OR Theo Von Ct[Title/Abstract]) OR Ct, Theo Von[Title/Abstract]) OR Von Ct, Theo[Title/Abstract]) OR Theo-24[Title/Abstract]) OR Theo 24[Title/Abstract]) OR Theo24[Title/Abstract]) OR Theobid[Title/Abstract]) OR Theocin[Title/Abstract]) OR Theoconfin Continuous[Title/Abstract]) OR Theodur[Title/Abstract]) OR Theo-Dur[Title/Abstract]) OR Theo Dur[Title/Abstract]) OR Theolair[Title/Abstract]) OR Theolix[Title/Abstract]) OR Theon[Title/Abstract]) OR Aerolate[Title/Abstract]) OR Theonite[Title/Abstract]) OR Theopek[Title/Abstract]) OR Theophylline Sodium Glycinate[Title/Abstract]) OR Glycinate, Theophylline Sodium[Title/Abstract]) OR Sodium Glycinate, Theophylline[Title/Abstract]) OR Theospan[Title/Abstract]) OR Theostat[Title/Abstract]) OR Theovent[Title/Abstract]) OR Uniphyl[Title/Abstract]) OR Uniphylline[Title/Abstract]) OR Theophylline Anhydrous[Title/Abstract]) OR Anhydrous, Theophylline[Title/Abstract]) OR Accurbron[Title/Abstract]) OR Aerobin[Title/Abstract]) OR Afonilum Retard[Title/Abstract]) OR Aquaphyllin[Title/Abstract]) OR Bronchoparat[Title/Abstract]) OR Bronkodyl[Title/Abstract])) OR "Aminophylline"[Mesh]) OR (((((((((((((((((((((((((((((((((Theophylline Ethylenediamine[Title/Abstract]) OR Ethylenediamine, Theophylline[Title/Abstract]) OR Theophyllamine[Title/Abstract]) OR Phyllotemp[Title/Abstract]) OR Mundiphyllin[Title/Abstract]) OR Theophyllamin Jenapharm[Title/Abstract]) OR Theophyllin EDA-ratiopharm[Title/Abstract]) OR Theophyllin EDA ratiopharm[Title/Abstract]) OR Theophyllin EDAratiopharm[Title/Abstract]) OR Truphylline[Title/Abstract]) OR Afonilum[Title/Abstract]) OR Carine[Title/Abstract]) OR Eufilina[Title/Abstract]) OR Euphyllin[Title/Abstract]) OR Aminodur[Title/Abstract]) OR Aminophyllin[Title/Abstract]) OR Aminophylline DF[Title/Abstract]) OR Cardophyllin[Title/Abstract]) OR Clonofilin[Title/Abstract]) OR Corophyllin[Title/Abstract]) OR Diaphyllin[Title/Abstract]) OR Drafilyn[Title/Abstract]) OR Duraphyllin[Title/Abstract]) OR Eufilina Venosa[Title/Abstract]) OR Euphyllin Retard[Title/Abstract]) OR Euphylline[Title/Abstract]) OR Godafilin[Title/Abstract]) OR Mini-Lix[Title/Abstract]) OR Mundiphyllin Retard[Title/Abstract]) OR Novophyllin[Title/Abstract]) OR Phyllocontin[Title/Abstract]) OR Somophyllin[Title/Abstract]) OR Tari-Dog[Title/Abstract]))) AND (((((Hormones, Adrenal Cortex[Title/Abstract]) OR Corticosteroids[Title/Abstract]) OR Corticoids[Title/Abstract])) OR "Adrenal Cortex Hormones"[Mesh])) AND (((("Lung Diseases, Obstructive"[Mesh]) OR (((((((Lung Disease, Obstructive[Title/Abstract]) OR Obstructive Lung Disease[Title/Abstract]) OR Obstructive Lung Diseases[Title/Abstract]) OR Obstructive Pulmonary Diseases[Title/Abstract]) OR Obstructive Pulmonary Disease[Title/Abstract]) OR Pulmonary Disease, Obstructive[Title/Abstract]) OR Pulmonary Diseases, Obstructive[Title/Abstract]))) OR (((((((((((COPD[Title/Abstract]) OR Chronic Obstructive Pulmonary Disease[Title/Abstract]) OR COAD[Title/Abstract]) OR Chronic Obstructive Airway Disease[Title/Abstract]) OR Chronic Obstructive Lung Disease[Title/Abstract]) OR Airflow Obstruction, Chronic[Title/Abstract]) OR Airflow Obstructions, Chronic[Title/Abstract]) OR Chronic Airflow Obstructions[Title/Abstract]) OR Chronic Airflow Obstruction[Title/Abstract])) OR "Pulmonary Disease, Chronic Obstructive"[Mesh]))** | [1166](https://www.ncbi.nlm.nih.gov/pubmed/?cmd=HistorySearch&querykey=53) | 05:16:34 |
| [#52](https://www.ncbi.nlm.nih.gov/pubmed/advanced) | [Add](https://www.ncbi.nlm.nih.gov/pubmed/advanced) | Search **((("Theophylline"[Mesh]) OR (((((((((((((((((((((((((((((((((((((((((((((((((((((((((((((3,7-Dihydro-1,3-dimethyl-1H-purine-2,6-dione[Title/Abstract]) OR 1,3-Dimethylxanthine[Title/Abstract]) OR 1,3 Dimethylxanthine[Title/Abstract]) OR Armophylline[Title/Abstract]) OR Constant-T[Title/Abstract]) OR Constant T[Title/Abstract]) OR ConstantT[Title/Abstract]) OR Elixophyllin[Title/Abstract]) OR Euphylong[Title/Abstract]) OR Glycine Theophyllinate[Title/Abstract]) OR Theophyllinate, Glycine[Title/Abstract]) OR Lodrane[Title/Abstract]) OR Uniphyllin[Title/Abstract]) OR Nuelin S.A.[Title/Abstract]) OR Nuelin[Title/Abstract]) OR Monospan[Title/Abstract]) OR Quibron T-SR[Title/Abstract]) OR Quibron T SR[Title/Abstract]) OR Quibron TSR[Title/Abstract]) OR Slo-Phyllin[Title/Abstract]) OR Slo Phyllin[Title/Abstract]) OR SloPhyllin[Title/Abstract]) OR Somophyllin-T[Title/Abstract]) OR Somophyllin T[Title/Abstract]) OR SomophyllinT[Title/Abstract]) OR Sustaire[Title/Abstract]) OR Synophylate[Title/Abstract]) OR Theo Von Ct[Title/Abstract]) OR Ct, Theo Von[Title/Abstract]) OR Von Ct, Theo[Title/Abstract]) OR Theo-24[Title/Abstract]) OR Theo 24[Title/Abstract]) OR Theo24[Title/Abstract]) OR Theobid[Title/Abstract]) OR Theocin[Title/Abstract]) OR Theoconfin Continuous[Title/Abstract]) OR Theodur[Title/Abstract]) OR Theo-Dur[Title/Abstract]) OR Theo Dur[Title/Abstract]) OR Theolair[Title/Abstract]) OR Theolix[Title/Abstract]) OR Theon[Title/Abstract]) OR Aerolate[Title/Abstract]) OR Theonite[Title/Abstract]) OR Theopek[Title/Abstract]) OR Theophylline Sodium Glycinate[Title/Abstract]) OR Glycinate, Theophylline Sodium[Title/Abstract]) OR Sodium Glycinate, Theophylline[Title/Abstract]) OR Theospan[Title/Abstract]) OR Theostat[Title/Abstract]) OR Theovent[Title/Abstract]) OR Uniphyl[Title/Abstract]) OR Uniphylline[Title/Abstract]) OR Theophylline Anhydrous[Title/Abstract]) OR Anhydrous, Theophylline[Title/Abstract]) OR Accurbron[Title/Abstract]) OR Aerobin[Title/Abstract]) OR Afonilum Retard[Title/Abstract]) OR Aquaphyllin[Title/Abstract]) OR Bronchoparat[Title/Abstract]) OR Bronkodyl[Title/Abstract])) OR "Aminophylline"[Mesh]) OR (((((((((((((((((((((((((((((((((Theophylline Ethylenediamine[Title/Abstract]) OR Ethylenediamine, Theophylline[Title/Abstract]) OR Theophyllamine[Title/Abstract]) OR Phyllotemp[Title/Abstract]) OR Mundiphyllin[Title/Abstract]) OR Theophyllamin Jenapharm[Title/Abstract]) OR Theophyllin EDA-ratiopharm[Title/Abstract]) OR Theophyllin EDA ratiopharm[Title/Abstract]) OR Theophyllin EDAratiopharm[Title/Abstract]) OR Truphylline[Title/Abstract]) OR Afonilum[Title/Abstract]) OR Carine[Title/Abstract]) OR Eufilina[Title/Abstract]) OR Euphyllin[Title/Abstract]) OR Aminodur[Title/Abstract]) OR Aminophyllin[Title/Abstract]) OR Aminophylline DF[Title/Abstract]) OR Cardophyllin[Title/Abstract]) OR Clonofilin[Title/Abstract]) OR Corophyllin[Title/Abstract]) OR Diaphyllin[Title/Abstract]) OR Drafilyn[Title/Abstract]) OR Duraphyllin[Title/Abstract]) OR Eufilina Venosa[Title/Abstract]) OR Euphyllin Retard[Title/Abstract]) OR Euphylline[Title/Abstract]) OR Godafilin[Title/Abstract]) OR Mini-Lix[Title/Abstract]) OR Mundiphyllin Retard[Title/Abstract]) OR Novophyllin[Title/Abstract]) OR Phyllocontin[Title/Abstract]) OR Somophyllin[Title/Abstract]) OR Tari-Dog[Title/Abstract])** | [388734](https://www.ncbi.nlm.nih.gov/pubmed/?cmd=HistorySearch&querykey=52) | 05:15:30 |
| [#51](https://www.ncbi.nlm.nih.gov/pubmed/advanced) | [Add](https://www.ncbi.nlm.nih.gov/pubmed/advanced) | Search **((((((((((((((((((((((((((((((((Theophylline Ethylenediamine[Title/Abstract]) OR Ethylenediamine, Theophylline[Title/Abstract]) OR Theophyllamine[Title/Abstract]) OR Phyllotemp[Title/Abstract]) OR Mundiphyllin[Title/Abstract]) OR Theophyllamin Jenapharm[Title/Abstract]) OR Theophyllin EDA-ratiopharm[Title/Abstract]) OR Theophyllin EDA ratiopharm[Title/Abstract]) OR Theophyllin EDAratiopharm[Title/Abstract]) OR Truphylline[Title/Abstract]) OR Afonilum[Title/Abstract]) OR Carine[Title/Abstract]) OR Eufilina[Title/Abstract]) OR Euphyllin[Title/Abstract]) OR Aminodur[Title/Abstract]) OR Aminophyllin[Title/Abstract]) OR Aminophylline DF[Title/Abstract]) OR Cardophyllin[Title/Abstract]) OR Clonofilin[Title/Abstract]) OR Corophyllin[Title/Abstract]) OR Diaphyllin[Title/Abstract]) OR Drafilyn[Title/Abstract]) OR Duraphyllin[Title/Abstract]) OR Eufilina Venosa[Title/Abstract]) OR Euphyllin Retard[Title/Abstract]) OR Euphylline[Title/Abstract]) OR Godafilin[Title/Abstract]) OR Mini-Lix[Title/Abstract]) OR Mundiphyllin Retard[Title/Abstract]) OR Novophyllin[Title/Abstract]) OR Phyllocontin[Title/Abstract]) OR Somophyllin[Title/Abstract]) OR Tari-Dog[Title/Abstract]** | [1400](https://www.ncbi.nlm.nih.gov/pubmed/?cmd=HistorySearch&querykey=51) | 05:15:04 |
| [#50](https://www.ncbi.nlm.nih.gov/pubmed/advanced) | [Add](https://www.ncbi.nlm.nih.gov/pubmed/advanced) | Search **"Aminophylline"[Mesh]** | [4183](https://www.ncbi.nlm.nih.gov/pubmed/?cmd=HistorySearch&querykey=50) | 05:08:29 |
| [#47](https://www.ncbi.nlm.nih.gov/pubmed/advanced) | [Add](https://www.ncbi.nlm.nih.gov/pubmed/advanced) | Search **((((((((((((((((((((((((((((((((((((((((((((((((((((((((((((3,7-Dihydro-1,3-dimethyl-1H-purine-2,6-dione[Title/Abstract]) OR 1,3-Dimethylxanthine[Title/Abstract]) OR 1,3 Dimethylxanthine[Title/Abstract]) OR Armophylline[Title/Abstract]) OR Constant-T[Title/Abstract]) OR Constant T[Title/Abstract]) OR ConstantT[Title/Abstract]) OR Elixophyllin[Title/Abstract]) OR Euphylong[Title/Abstract]) OR Glycine Theophyllinate[Title/Abstract]) OR Theophyllinate, Glycine[Title/Abstract]) OR Lodrane[Title/Abstract]) OR Uniphyllin[Title/Abstract]) OR Nuelin S.A.[Title/Abstract]) OR Nuelin[Title/Abstract]) OR Monospan[Title/Abstract]) OR Quibron T-SR[Title/Abstract]) OR Quibron T SR[Title/Abstract]) OR Quibron TSR[Title/Abstract]) OR Slo-Phyllin[Title/Abstract]) OR Slo Phyllin[Title/Abstract]) OR SloPhyllin[Title/Abstract]) OR Somophyllin-T[Title/Abstract]) OR Somophyllin T[Title/Abstract]) OR SomophyllinT[Title/Abstract]) OR Sustaire[Title/Abstract]) OR Synophylate[Title/Abstract]) OR Theo Von Ct[Title/Abstract]) OR Ct, Theo Von[Title/Abstract]) OR Von Ct, Theo[Title/Abstract]) OR Theo-24[Title/Abstract]) OR Theo 24[Title/Abstract]) OR Theo24[Title/Abstract]) OR Theobid[Title/Abstract]) OR Theocin[Title/Abstract]) OR Theoconfin Continuous[Title/Abstract]) OR Theodur[Title/Abstract]) OR Theo-Dur[Title/Abstract]) OR Theo Dur[Title/Abstract]) OR Theolair[Title/Abstract]) OR Theolix[Title/Abstract]) OR Theon[Title/Abstract]) OR Aerolate[Title/Abstract]) OR Theonite[Title/Abstract]) OR Theopek[Title/Abstract]) OR Theophylline Sodium Glycinate[Title/Abstract]) OR Glycinate, Theophylline Sodium[Title/Abstract]) OR Sodium Glycinate, Theophylline[Title/Abstract]) OR Theospan[Title/Abstract]) OR Theostat[Title/Abstract]) OR Theovent[Title/Abstract]) OR Uniphyl[Title/Abstract]) OR Uniphylline[Title/Abstract]) OR Theophylline Anhydrous[Title/Abstract]) OR Anhydrous, Theophylline[Title/Abstract]) OR Accurbron[Title/Abstract]) OR Aerobin[Title/Abstract]) OR Afonilum Retard[Title/Abstract]) OR Aquaphyllin[Title/Abstract]) OR Bronchoparat[Title/Abstract]) OR Bronkodyl[Title/Abstract]** | [361630](https://www.ncbi.nlm.nih.gov/pubmed/?cmd=HistorySearch&querykey=47) | 05:07:15 |
| [#46](https://www.ncbi.nlm.nih.gov/pubmed/advanced) | [Add](https://www.ncbi.nlm.nih.gov/pubmed/advanced) | Search **"Theophylline"[Mesh]** | [28039](https://www.ncbi.nlm.nih.gov/pubmed/?cmd=HistorySearch&querykey=46) | 04:54:22 |
| [#43](https://www.ncbi.nlm.nih.gov/pubmed/advanced) | [Add](https://www.ncbi.nlm.nih.gov/pubmed/advanced) | Search **((((Hormones, Adrenal Cortex[Title/Abstract]) OR Corticosteroids[Title/Abstract]) OR Corticoids[Title/Abstract])) OR "Adrenal Cortex Hormones"[Mesh]** | [318906](https://www.ncbi.nlm.nih.gov/pubmed/?cmd=HistorySearch&querykey=43) | 04:50:24 |
| [#42](https://www.ncbi.nlm.nih.gov/pubmed/advanced) | [Add](https://www.ncbi.nlm.nih.gov/pubmed/advanced) | Search **((Hormones, Adrenal Cortex[Title/Abstract]) OR Corticosteroids[Title/Abstract]) OR Corticoids[Title/Abstract]** | [84067](https://www.ncbi.nlm.nih.gov/pubmed/?cmd=HistorySearch&querykey=42) | 04:50:13 |
| [#41](https://www.ncbi.nlm.nih.gov/pubmed/advanced) | [Add](https://www.ncbi.nlm.nih.gov/pubmed/advanced) | Search **"Adrenal Cortex Hormones"[Mesh]** | [277942](https://www.ncbi.nlm.nih.gov/pubmed/?cmd=HistorySearch&querykey=41) | 04:49:30 |
| [#38](https://www.ncbi.nlm.nih.gov/pubmed/advanced) | [Add](https://www.ncbi.nlm.nih.gov/pubmed/advanced) | Search **((("Lung Diseases, Obstructive"[Mesh]) OR (((((((Lung Disease, Obstructive[Title/Abstract]) OR Obstructive Lung Disease[Title/Abstract]) OR Obstructive Lung Diseases[Title/Abstract]) OR Obstructive Pulmonary Diseases[Title/Abstract]) OR Obstructive Pulmonary Disease[Title/Abstract]) OR Pulmonary Disease, Obstructive[Title/Abstract]) OR Pulmonary Diseases, Obstructive[Title/Abstract]))) OR (((((((((((COPD[Title/Abstract]) OR Chronic Obstructive Pulmonary Disease[Title/Abstract]) OR COAD[Title/Abstract]) OR Chronic Obstructive Airway Disease[Title/Abstract]) OR Chronic Obstructive Lung Disease[Title/Abstract]) OR Airflow Obstruction, Chronic[Title/Abstract]) OR Airflow Obstructions, Chronic[Title/Abstract]) OR Chronic Airflow Obstructions[Title/Abstract]) OR Chronic Airflow Obstruction[Title/Abstract])) OR "Pulmonary Disease, Chronic Obstructive"[Mesh])** | [230543](https://www.ncbi.nlm.nih.gov/pubmed/?cmd=HistorySearch&querykey=38) | 04:46:37 |
| [#37](https://www.ncbi.nlm.nih.gov/pubmed/advanced) | [Add](https://www.ncbi.nlm.nih.gov/pubmed/advanced) | Search **("Lung Diseases, Obstructive"[Mesh]) OR (((((((Lung Disease, Obstructive[Title/Abstract]) OR Obstructive Lung Disease[Title/Abstract]) OR Obstructive Lung Diseases[Title/Abstract]) OR Obstructive Pulmonary Diseases[Title/Abstract]) OR Obstructive Pulmonary Disease[Title/Abstract]) OR Pulmonary Disease, Obstructive[Title/Abstract]) OR Pulmonary Diseases, Obstructive[Title/Abstract])** | [226300](https://www.ncbi.nlm.nih.gov/pubmed/?cmd=HistorySearch&querykey=37) | 04:46:06 |
| [#36](https://www.ncbi.nlm.nih.gov/pubmed/advanced) | [Add](https://www.ncbi.nlm.nih.gov/pubmed/advanced) | Search **((((((Lung Disease, Obstructive[Title/Abstract]) OR Obstructive Lung Disease[Title/Abstract]) OR Obstructive Lung Diseases[Title/Abstract]) OR Obstructive Pulmonary Diseases[Title/Abstract]) OR Obstructive Pulmonary Disease[Title/Abstract]) OR Pulmonary Disease, Obstructive[Title/Abstract]) OR Pulmonary Diseases, Obstructive[Title/Abstract]** | [60355](https://www.ncbi.nlm.nih.gov/pubmed/?cmd=HistorySearch&querykey=36) | 04:45:37 |
| [#35](https://www.ncbi.nlm.nih.gov/pubmed/advanced) | [Add](https://www.ncbi.nlm.nih.gov/pubmed/advanced) | Search **"Lung Diseases, Obstructive"[Mesh]** | [204062](https://www.ncbi.nlm.nih.gov/pubmed/?cmd=HistorySearch&querykey=35) | 04:44:05 |
| [#33](https://www.ncbi.nlm.nih.gov/pubmed/advanced) | [Add](https://www.ncbi.nlm.nih.gov/pubmed/advanced) | Search **((((((((((COPD[Title/Abstract]) OR Chronic Obstructive Pulmonary Disease[Title/Abstract]) OR COAD[Title/Abstract]) OR Chronic Obstructive Airway Disease[Title/Abstract]) OR Chronic Obstructive Lung Disease[Title/Abstract]) OR Airflow Obstruction, Chronic[Title/Abstract]) OR Airflow Obstructions, Chronic[Title/Abstract]) OR Chronic Airflow Obstructions[Title/Abstract]) OR Chronic Airflow Obstruction[Title/Abstract])) OR "Pulmonary Disease, Chronic Obstructive"[Mesh]** | [82499](https://www.ncbi.nlm.nih.gov/pubmed/?cmd=HistorySearch&querykey=33) | 04:42:53 |
| [#32](https://www.ncbi.nlm.nih.gov/pubmed/advanced) | [Add](https://www.ncbi.nlm.nih.gov/pubmed/advanced) | Search **((((((((COPD[Title/Abstract]) OR Chronic Obstructive Pulmonary Disease[Title/Abstract]) OR COAD[Title/Abstract]) OR Chronic Obstructive Airway Disease[Title/Abstract]) OR Chronic Obstructive Lung Disease[Title/Abstract]) OR Airflow Obstruction, Chronic[Title/Abstract]) OR Airflow Obstructions, Chronic[Title/Abstract]) OR Chronic Airflow Obstructions[Title/Abstract]) OR Chronic Airflow Obstruction[Title/Abstract]** | [62662](https://www.ncbi.nlm.nih.gov/pubmed/?cmd=HistorySearch&querykey=32) | 04:42:36 |
| [#31](https://www.ncbi.nlm.nih.gov/pubmed/advanced) | [Add](https://www.ncbi.nlm.nih.gov/pubmed/advanced) | Search **"Pulmonary Disease, Chronic Obstructive"[Mesh]** | [52427](https://www.ncbi.nlm.nih.gov/pubmed/?cmd=HistorySearch&querykey=31) | 04:40:10 |

**WEB OF SCIENCE**

| # 9 | [**373**](http://apps.webofknowledge.com/summary.do?product=UA&doc=1&qid=11&SID=8BRfQp1SiLXHiphAEpa&search_mode=CombineSearches&update_back2search_link_param=yes) | #8 AND #2 AND #1 |  |  |
| --- | --- | --- | --- | --- |
| # 8 | [**163,657**](http://apps.webofknowledge.com/summary.do?product=UA&doc=1&qid=10&SID=8BRfQp1SiLXHiphAEpa&search_mode=CombineSearches&update_back2search_link_param=yes) | #7 OR #6 OR #5 OR #4 OR #3 |  |  |
| # 7 | [**279**](http://apps.webofknowledge.com/summary.do?product=UA&doc=1&qid=9&SID=8BRfQp1SiLXHiphAEpa&search_mode=GeneralSearch&update_back2search_link_param=yes) | **TS:** (Truphylline) *OR* **TS:** (Afonilum) *OR* **TS:** (Carine) *OR* **TS:** (Eufilina) *OR* **TS:** (Euphyllin) *OR* **TS:** (Aminodur) *OR* **TS:** (Aminophyllin) *OR***TS:** (Aminophylline DF) *OR* **TS:** (Cardophyllin) *OR* **TS:** (Clonofilin) *OR* **TS:** (Corophyllin) *OR* **TS:** (Diaphyllin) *OR* **TS:** (Drafilyn) *OR* **TS:**(Duraphyllin) *OR* **TS:** (Eufilina Venosa) *OR* **TS:** (Euphyllin Retard) *OR* **TS:** (Euphylline) *OR* **TS:** (Godafilin) *OR* **TS:** (Mini-Lix) *OR* **TS:**(Mundiphyllin Retard) *OR* **TS:** (Novophyllin) *OR* **TS:** (Phyllocontin) *OR* **TS:** (Somophyllin) *OR* **TS:** (Tari-Dog) |  |  |
| # 5 | [**440**](http://apps.webofknowledge.com/summary.do?product=UA&doc=1&qid=5&SID=8BRfQp1SiLXHiphAEpa&search_mode=GeneralSearch&update_back2search_link_param=yes) | **TS:** (Theoconfin Continuous) *OR* **TS:** (Theodur) *OR* **TS:** (Theo-Dur) *OR* **TS:** (Theo Dur) *OR* **TS:** (Theolair) *OR* **TS:** (Theolix) *OR* **TS:** (Theon) *OR* **TS:** (Aerolate) *OR* **TS:** (Theonite) *OR* **TS:** (Theopek) *OR* **TS:** (Theophylline Sodium Glycinate) *OR* **TS:** (Glycinate, Theophylline Sodium) *OR***TS:** (Sodium Glycinate, Theophylline) *OR* **TS:** (Theospan) *OR* **TS:** (Theostat) *OR* **TS:** (Theovent) *OR* **TS:** (Uniphyl) *OR* **TS:** (Uniphylline) |  |  |
| # 4 | [**174**](http://apps.webofknowledge.com/summary.do?product=UA&doc=1&qid=4&SID=8BRfQp1SiLXHiphAEpa&search_mode=GeneralSearch&update_back2search_link_param=yes) | **TS:** (Quibron T SR) *OR* **TS:** (Quibron TSR) *OR* **TS:** (Slo-Phyllin) *OR* **TS:** (Slo Phyllin) *OR* **TS:** (SloPhyllin) *OR* **TS:** (Somophyllin-T) *OR* **TS:**(Somophyllin T) *OR* **TS:** (SomophyllinT) *OR* **TS:** (Sustaire) *OR* **TS:** (Synophylate) *OR* **TS:** (Theo Von Ct) *OR* **TS:** (Ct, Theo Von) *OR* **TS:** (Von Ct, Theo) *OR* **TS:** (Theo-24) *OR* **TS:** (Theo 24) *OR* **TS:** (Theo24) *OR* **TS:** (Theobid) *OR* **TS:** (Theocin) |  |  |
| # 3 | [**160,642**](http://apps.webofknowledge.com/summary.do?product=UA&doc=1&qid=3&SID=8BRfQp1SiLXHiphAEpa&search_mode=GeneralSearch&update_back2search_link_param=yes) | **TS:** (Theophylline) *OR* **TS:** (3,7-Dihydro-1,3-dimethyl-1H-purine-2,6-dione) *OR* **TS:** (1,3-Dimethylxanthine) *OR* **TS:** (1,3 Dimethylxanthine) *OR* **TS:** (Armophylline) *OR* **TS:** (Constant-T) *OR* **TS:** (Constant T) *OR* **TS:** (ConstantT) *OR* **TS:** (Elixophyllin) *OR***TS:** (Euphylong) *OR* **TS:** (Glycine Theophyllinate) *OR* **TS:** (Theophyllinate, Glycine) *OR* **TS:** (Lodrane) *OR* **TS:** (Uniphyllin) *OR* **TS:** (Nuelin S.A.) *OR* **TS:** (Nuelin) *OR* **TS:** (Monospan) *OR* **TS:** (Quibron T-SR) |  |  |
| # 2 | [**142,901**](http://apps.webofknowledge.com/summary.do?product=UA&doc=1&qid=2&SID=8BRfQp1SiLXHiphAEpa&search_mode=GeneralSearch&update_back2search_link_param=yes) | **TS:** (Adrenal Cortex Hormones) *OR* **TS:** (Hormones, Adrenal Cortex) *OR* **TS:** (Corticosteroids) *OR* **TS:** (Corticoids) |  |  |
| # 1 | [**123,040**](http://apps.webofknowledge.com/summary.do?product=UA&doc=1&qid=1&SID=8BRfQp1SiLXHiphAEpa&search_mode=GeneralSearch&update_back2search_link_param=yes) | **TS:** (Pulmonary Disease, Chronic Obstructive) *OR* **TS:** (COPD) *OR* **TS:** (Chronic Obstructive Pulmonary Disease) *OR* **TS:** (COAD) *OR* **TS:**(Chronic Obstructive Airway Disease) *OR* **TS:** (Chronic Obstructive Lung Disease) *OR* **TS:** (Airflow Obstruction, Chronic) *OR* **TS:** (Airflow Obstructions, Chronic) *OR* **TS:** (Chronic Airflow Obstructions) *OR* **TS:** (Chronic Airflow Obstruction) *OR* **TS:** (Lung Diseases, Obstructive) *OR* **TS:**(Lung Disease, Obstructive) *OR* **TS:** (Obstructive Lung Disease) *OR* **TS:** (Obstructive Lung Diseases) *OR* **TS:** (Obstructive Pulmonary Diseases) *OR***TS:** (Obstructive Pulmonary Disease) *OR* **TS:** (Pulmonary Disease, Obstructive) *OR* **TS:** (Pulmonary Diseases, Obstructive) |  |  |
