# Supplementary material for: Low-dose theophylline in addition to ICS therapy in COPD patients: A systematic review and meta-analysis
Source: PLoS One. 2021 May 24;16(5):e0251348. doi: 10.1371/journal.pone.0251348 (PMC8143407; doi:10.1371/journal.pone.0251348)
Supplement: S3 File — (PDF) [file pone.0251348.s007.pdf]

Adding theophylline to ICS therapy on COPD: a protocol for systematic review and meta-analysis

Tiankui Shuai, Lufang Feng, Liping Liu, Chuchu Zhang, Jian Liu

To enable PROSPERO to focus on COVID-19 registrations during the 2020 pandemic, this registration record was automatically published exactly as submitted. The PROSPERO team has not checked eligibility.

Citation

Tiankui Shuai, Lufang Feng, Liping Liu, Chuchu Zhang, Jian Liu. Adding theophylline to ICS therapy on COPD: a protocol for systematic review and meta-analysis. PROSPERO 2021 CRD42021224952 Available from:
https://www.crd.york.ac.uk/prospero/display_record.php?ID=CRD42021224952

Review question

To study the efficacy and safety of adding theophylline to ICS therapy in the treatment of COPD patients

Searches

PubMed, Web of Science, Cochrane Library, Embase

Types of study to be included

Any comparative study, i.e., cohort, case-control, RCTs

Condition or domain being studied

Chronic obstructive pulmonary disease (COPD) is a common and frequently occurring respiratory disease. A preventable and treatable disease characterized by limited airflow, associated with abnormal inflammatory responses of the lungs to harmful particles or gases, and COPD may also affect changes in various systems throughout the body, thus having systemic effects.

Participants/population

Patients with chronic obstructive pulmonary disease

Intervention(s), exposure(s)

theophylline to ICS therapy

Comparator(s)/control

ICS therapy

Main outcome(s)

Severe COPD exacerbations, COPD hospital admissions, health-related quality-of-life score, total admissions, FEV1, FVC, MVV, CAT, mMRC, pneumonia, all-cause mortality, COPD-related mortality and adverse effects.

* Measures of effect

Mean difference, odds ratio (OR) or relative risks (RR)

Additional outcome(s)

None

* Measures of effect

None

Data extraction (selection and coding)

Author, publication year, country, study design, characteristics of participants(i.e., age[MD±SD], sex), treatment period, dosage, sample size(n, cases, control), effect size(WMD, SMD, RR, OR, HR with their 95%CI), outcome indicator value(i.e., exacerbations, FEV1, FVC, MVV, CAT, mMRC, pneumonia, mortality, adverse effects, mortality).

Risk of bias (quality) assessment

Cochrane collaboration's tool forassessing risk of bias, NOS scale(cohort or case-control study), AHQR scale(cross-sectional study)

Strategy for data synthesis

We calculated the results of individual studies as fixed eHect weighted mean diference (WMD) or standardised mean diference (SMD) including the 95% confidence interval (CI) for each outcome. Where results were expressed as dichotomous variables, we calculated odds ratio (OR) or relative risks (RR) with 95% CI for individual outcomes. Stata/SE 15.0 software was used for statistical analyses. Heterogeneity between studies was assessed using the Higgins I^2 test and P values, with $P < 0.05$ indicating the significant heterogeneity between studies, and the I^2 statistic which describes the percentage of total variation across trials that is due to heterogeneity rather than sampling error. The values of 25%, 50%, and 75% for the I^2 as indicative of low, moderate, and high statistical heterogeneity, respectively. If $P < 0.05$ and $I^2 > 50\%$, the random effects model was selected to calculate the pooled effective size. Publication bias was assessed with funnel plots and sensitivity analyses were performed for studies that have a greater impact on the results. All tests were two tailed and $P < 0.05$ was considered significant.

Analysis of subgroups or subsets

When heterogeneity is high and data is supported, we will consider doing subgroup analysis, such as, country, study design, dosage.

Contact details for further information

Lufang Feng
898964127@qq.com

Organisational affiliation of the review

Lanzhou university

Review team members and their organisational affiliations

Mr Tiankui Shuai. Department of Intensive Care Unit, The First Hospital of Lanzhou University
Miss Lufang Feng. lanzhou university
Professor Liping Liu. Department of Intensive Care Unit, The First Hospital of Lanzhou University
Miss Chuchu Zhang. The First Clinical Medical College of the First Hospital of Lanzhou University
Professor Jian Liu. Department of Intensive Care Unit, The First Hospital of Lanzhou University

Type and method of review

Intervention, Meta-analysis, Systematic review

Anticipated or actual start date

20 December 2020

Anticipated completion date

01 June 2021

Funding sources/sponsors

No

Conflicts of interest

Language

English

Country

China

Stage of review
Review Completed published

Subject index terms status
Subject indexing assigned by CRD

Subject index terms
MeSH headings have not been applied to this record

Date of registration in PROSPERO
13 January 2021

Date of first submission
13 December 2020

Stage of review at time of this submission

Stage	Started	Completed
Preliminary searches	Yes	No
Piloting of the study selection process	Yes	No
Formal screening of search results against eligibility criteria	Yes	No
Data extraction	No	No
Risk of bias (quality) assessment	No	No
Data analysis	No	No

The record owner confirms that the information they have supplied for this submission is accurate and complete and they understand that deliberate provision of inaccurate information or omission of data may be construed as scientific misconduct.

The record owner confirms that they will update the status of the review when it is completed and will add publication details in due course.

Versions
13 January 2021
13 January 2021

PROSPERO
This information has been provided by the named contact for this review. CRD has accepted this information in good faith and registered the review in PROSPERO. The registrant confirms that the information supplied for this submission is accurate and complete. CRD bears no responsibility or liability for the content of this registration record, any associated files or external websites.
